# Tri-State Medical Society of Alabama, Georgia and Tennessee

**Published:** 1896-12

**Authors:** 


### TRI-STATE MEDICAL SOCIETY OF ALABAMA, GEORGIA AND TENNESSEE.

EIGHTH ANNUAL MEETING, HELD IN CHATTANOOGA, OCTOBER 13, 14 AND 15, 1896.

The president, Dr. J. B. Murfree, called the meeting to order, and the first paper read was by Dr. Y. L. Abernathy entitled: "Convulsions in Children Treated with Large Doses of Morphine." He cited several cases which were about to die, recovered under large doses of morphine.

Dr. Andrew Boyd had used large doses of morphine in cholera infantum as a dernier resort. In colvulsions and cholera infantum the cause seems to be entirely reflex and the treatment seems to be correct and rational no matter how heroic it may seem. He related a case where an infant six months old took 70 drops tr. opii in two and a half hours and four quarter-grain injections of morphine in six hours.

Dr. W. G. Bogart said that he understood that this was not the only treatment the doctor gave, but this with other treatment. He would give an active cathartic, a hot bath, a copious injection of hot water per rectum, and morphia, if the convulsions still continued, in doses large enough to control the convulsions.

Dr. D. S. Middleton read a paper on "Cystitis: Report of Cases," being a statistical report showing its frequency in women, a discussion of the pathology, cause (microdic infection), the treatment by antiseptic injections and report of cases.

Dr. G. A. Baxter would add only one remedy, a saturated solution of acetate of aluminum. Nitrate of silver sometimes produces tenesmus, and he no longer uses it.

Dr. R. R. Kime read: "Some Obstetrical Complications, with Report of Cases." (See November issue.)

Dr. W. G. Bogart indorsed most heartily the position taken by the writer. He said, "the first case was one we often meet with and we fail to appreciate the importance of a thorough drainage and antiseptic. The removal of all the foreign matter and a thorough washing out of the uterus with a hot antiseptic fluid and free drainage will generally result in recovery. Case 2 is one of rare occurrence—hemorrhage eight days after delivery. I desire to call attention to the manner in which the hemorrhage was controlled—packing with iodoform gauze and keeping it so until the hemorrhage ceased. The gauze also acted as a drain for the light fluid. I shall add that I would flush out the cavity with hot antiseptic solution. Case 3 is one of great danger, which demands knowledge, skill and immediate action. I heartily commend the doctor for his action, especially his manner of controlling the fever by antiseptics rather than antipyretics, followed by free drainage. I agree that tubular drainage is the only method of accomplishing the desired result and will almost invariably bring down the temperature. I condemn the use antipyretics, especially the coal tar derivatives. Cleanliness, drainage, alcoholic stimulants and quinine, I believe to be the rational treatment of these cases."

Dr. W. C. Bilbro reported "A Case of Bradycardia" from ptomaine poisoning. The history was one of indigestion. The pulse was 30 standing after walking two blocks, and was down to 18 at times. There were times when the pulse was 6, but regular. He laid stress on the apex beat. Improvement under arsenic, strychnia, etc. He divided bradycardia into two classes, physiological, as a constitutional peculiarity, and in the puerperal state; and pathological, as from exhaustion and in stomach affections.

Dr. J. R. Rathmell read a paper on "Scarlatina" and laid stress on the complications which are the cause of death and to which treatment should be directed. The contagion resides in the epidermal scales thrown off during desquamation, hence the necessity of isolation, especially during desquamation; also the need of a vigorous effort to destroy the almost imperishable germs hidden in the room, by fumigation, by boiling and even by burning all articles of clothing, bedding, etc., used during the attack, and a thorough renovation of walls and woodwork with kalsomining and paint in order to prevent a possible attack in future.

Dr. G. A. Baxter described "A New Splint for Fractures of the Humerus below the Surgical Neck," and demonstrated the same on a subject. It consists of a blunt wedge of tin for the axillary space to which is attached a right angle splint for the arm which is adjustible on humeral portion, allowing extension to be made and fixation before bandaging. The axilla is made the point of counter extension, extension from elbow. It allows examination in case of compound fractures without disturbing extension.

Dr. Baxter said that statistics showed as much as 33 per cent. of these fractures were ununited, and this splint was invented to meet the indications as found in his own cases. Fixation cannot be from shoulder, it must be from a fixed point. There will be no complaint from numbness of the fingers or pressure in the axilla. Any tinner can make the splint.

Dr. C. R. Achison read a paper: "Treatment of Cancer of the Skin," advocating the use of caustics in the treatment of epithelioma. There is much less destruction of tissue than with the knife. In the use of the latter if there are any cells left there will be a recurrence, while if the caustic is used the inflammation, etc., will cause the death of the pathologic cells beyond the point cauterized. The pain can be reduced to a minimum by mixing with cocaine or general anesthesia. Arsenious acid has a selective action, devitalizing the cancer cells. Caustic potash is specially useful on the lip. Chloride of zinc produces a dry slough.

Dr. W. Frank Glenn read a paper: "Diseases of the Veru Montanum (Caput Gallinaginis)." He described acute and chronic inflammation, hypertrophy, hyperesthesia, cause gonorrhea and masturbation, hyperesthesia especially due to masturbation. Symptoms: Frequent desire to urinate with dribbling at end of act.

Chronic inflammation most frequent cause of impotence. Internally he recommended alkalithia or maizi-lithium--locally argonin. Nitrate of silver should be avoided in acute cases. Recovery is slow. General tonics, galvanism, etc., will generally cure hypertrophies. In hyperesthesia there is premature ejaculation in the sexual act. The mistake here is that the case is often treated for sexual weakness with strychnia, phosphorus, etc., while the opposite course is indicated.

Dr. W. D. Haggard, Jr., read a paper on "Vaginal Hysterectomy for Bilateral Suppurative Processes of the Uterine Adnexa." He said that the reason for removing the uterus where the adnexa were hopelessly diseased, requiring removal, is founded on the following facts: A large number of cases where the tubes and ovaries were removed were not perfectly cured; the persistent symptom was pain; hysterectomy cured these cases. There were painful malpositions, a more stormy and protracted menopause. There was danger of adhesions to hollow viscera and subsequent obstruction; it takes no longer to do a total hysterectomy than curetting or ventro-fixation after double ovariotomy; the mortality is lower; the uterus is a part of the disease in pyogenic infection, hence hysterectomy was not the removal of a healthy, intact organ. The mortality in five hospitals was 18.5 per cent. in removal for tubes and ovaries alone for pus. Vaginal hysterectomy in 724 cases, 4.6 per cent.; Jacob's 403 cases, 2.9 per cent. The supreme triumph of the vaginal operation was that it afforded the means of a thorough exploration essential to conservative proceedure. The vaginal method preferable because: 1. The preliminary step, vaginal section, allows thorough exploration and conservative treatment, with a minimum of risk. 2. The vagina is the natural approach and logical avenue for drainage of the pelvis. 3. It is immune from the unpleasant sequelæ of laparotomy, possibility of hernia, stitch abscess, infected ligature and sinus, and the abdominal supporter. 4. Less immediate shock. Convalescence is smoother and shorter. 5. No exposure or handling of intestines. 6. Less danger of peritoneal contamination. 7. Mortality is lower. 8. Invades only the diseased area and leaves undisturbed the protecting mass of adhesions. The steps may be summarized as follows, but may be varied: 1. Preliminary curettage. 2. Completion of incision around cervix, prolonged transversely in the lateral fornices. 4. Freeing cervix anteriorly from the bladder and ureters. 5. Application of clamps to base of broad ligaments containing the uterine arteries. 6. Amputation of cervix. 7. Median section of the uterus. 8. Enucleation of each appendage separately. 9. Application of clamps to upper portion of broad ligaments containing ovarian arteries. 10. Excision of each lateral half of uterus with diseased mass."

Dr. W. E. B. Davis, read a paper on "Treatment of Pus in the Pelvis." He said that some of the French surgeons report inability to remove appendage in many cases per vaginam, but that the cases got well, demonstrating that drainage cures. This is the essential thing. Not necessary to remove uterus. High amputation of cervix, drain tubes, and curette uterus later. All cases will not be cured, but an abdominal section can be done later. In removing tubes it is better to go in from above as they are difficult to remove per vaginum.

Dr. J. A. Goggans opened the discussion on these two papers by saying that he followed the practice of Dr. Davis. He thought that we should be very conservative and seriously consider harmful sequelæ of complete ablation of genital organs in young women. Every appropriate treatment was justifiable when we consider the great variety of pathological conditions. He recognized three methods of treating pus in the pelvis: 1. Simple incision with drainage through vagina or abdomen. 2. Opening abscess by laparotomy. 3. Opening abscess per vaginam. Each applicable to suitable cases. He related a case of laparotomy drained finally through the vagina, followed by irrigations, recovery; also one of large pelvic abscess which ruptured during examination. An immediate laparotomy saved the patient.

Dr. W. F. Westmoreland read a paper: "Some Remarks on Syphilis," taking the ground that it was often communicated by means other than sexual intercourse: sixty cases infected from a catheter; a dozen children from vaccine point. Danger from doctors, from servants. Hereditary syphilis is quite common.

Dr. Westmoreland thought that we could do much to check syphilis. At his clinic there were many servants treated. Where there were lesions on the hands they were directed to quit work, and generally did so. He saw many cases where infection was not due to venery. In New York nine cases were reported of doctors who had the primary sore on the fingers. He would prevent the marriage of an innocent girl to a syphilitic; if necessary would inform the family of the girl, after telling the patient of his intention.

President J. B. Murfree delivered his annual address, "The Doctor of Medicine." He congratulated the society on its prosperity and urged greater efforts for its advancement. He advocated higher and more unselfish motives in the practice of medicine. He deprecated young men entering the profession simply to make money. Medicine is a poor trade for an honest man, and if money is the only object there are others that pay better. The doctor's first duty is to his patient, to give him his best services. He should be ethical, not allowing the love of gain or distinction to swerve him from the right. The reputation of a brother physician should be defended. He dwelt upon our duty to the public in regard to preventative medicine, and how to avoid quacks and patent medicines.

Dr. J. A. Goggans read a paper entitled: "A Few Unique Cases in Abdominal Surgery." He thought that surgeons should report their cases, especially the unsuccessful ones, that the science and art of surgery might be of greatest utility to humanity. He presented a specimen of ovaries that contained serous and myomatous multiple follicular cysts, removed from a woman 55 years old. Related a case of abdominal section for tubercular peritonitis. The patient had been an invalid seven months, temperature 101, pulse 110. More than one-half gallon of ascitic fluid was evacuated, abdominal cavity irrigated with normal salt solution. Patient made a good recovery from incision, and was able to work most of the time. The third case was that of a child. He presented a stomach and intestines removed *post mortem* with their mesentery. Diagnosis was impossible without exploratory incision. The fourth case was that of right hemiplegia following an exploratory incision in a female 60 years old. The patient was recovering from the paralysis. The fifth was the only case that has ever recovered after operation, in America, and perhaps the only case where operation has been undertaken for exactly that condition in this country. The patient had been out of health for two years, but her abdominal pains and enlargement had existed only eight or ten months. She had been treated for abdominal dropsy. On opening the abdomen the tumor proved to be one of the mesentery. He stitched and drained, and the patient recovered. The other successful cases were one operated on by Bantock, in London, and one by Pean, in Paris.

Dr. Catherine R. Collins read a paper entitled: "Microscopical and Chemical Aids to Diagnosis," dwelling on the importance of examinations of the urine. It should be examined for five days in succession. The whole amount passed in twenty-four hours saved. That in the morning gives the urine of a fasting patient. She related cases illustrating her position. She then called attention to the use of the microscope in diagnosing many diseases, especially tuberculosis, diphtheria and malaria, and also the changing views in regard to the origin of typhoid fever. The Eberth germ, according to Vaughan, not being the cause, but an involution form of one of several germs that may, separately or collectively, be the cause of the disease.

Dr. R. H. Hayes read: "A Statistical Report of the More Recent Remedies Used in the Treatment of Tuberculosis, and Summary of Recent Preventative Methods of Value," in which he gave an array of clinical and bacteriological statistics from such men as Koch, Maragliano, Campana, Kleis, Hunter and others in Europe, von Ruck, Taylor, Dennison and Vaughn, and others; also a report from a committee from the New Orleans Parish Medical Society in favor of the newer or biological, or directly germicidal remedies, originated by Koch, as tuberculin, tuberculocidin, antiphthisin; also the nuclein preparations of Vaughan, and such medicines as pilocarpin and aseptolin. These statistics, he claimed, were good evidence in favor of the remedies, as they were clinical, and were from men prepared to apply them properly, carefully, and who had taken the proper time. The use of these newer remedial agents in tuberculosis had reduced the general mortality rate perceptibly. He also gave an outline of the work of the New York Board of Health to restrict tuberculosis. This consisted in public and private instruction as to danger from milk and meat (bovine tuberculosis), and from dried sputum (human tuberculosis), it having been conceded that from these sources the large majority of cases were contracted. By thorough system of instruction the board had gotten better results in preventing spread of disease and had succeeded in largely reducing mortality rate from tuberculosis, and had demonstrated beyond doubt that it was contagious, infectious and preventable.

Dr. Y. L. Abernathy thought that there was nothing in serotherapy. The remedy was made to sell. Koch's tuberculin was a failure, but millions were made out of it before it was found out. Pasteur's hydrophobia and tetanus cures, Hammond's serums, Brown-Sequard's elixir of life, all on a par. They get up wonderful statistics. Something may be developed along these lines as good as vaccination, but it is still in the future. At present they are only fads, and very expensive and silly fads.

Dr. Geo. S. Brown said that we had the two extremes in the reader and the last speaker. Koch had done more than any other man to reduce the mortality from consumption, not from serum therapy, but because the disease was better understood. The principle has not been discovered that will cure consumption. Some who make great claims do not have the confidence of bacteriologists. When the true principle is discovered it will not require any great amount of advertising. The man may have his laboratory in Sahara desert, but will do a good business. In diphtheria the true principle has been found, and antitoxine is a success. The serum treatment offers much in the future.

The following officers were elected for the ensuing year:

President—Willis F. Westmoreland, Atlanta, Ga.

Vice-Presidents—M. B. Hutchins, Atlanta, Ga.; Seale Harris, Union Springs, Ala.; C. R. Achison, Nashville, Tenn.

Secretary—Frank Trester Smith, Chattanooga, Tenn.

Treasurer—Geo. R. West, Chattanooga, Tenn.

Chairman Committee of Arrangements—W. D. Haggard, Jr., Nashville, Tenn.

Adjourned to meet in Nashville on the second Tuesday in October, 1897.

